# Possible prediction of patterns of cervical lymph node spread based on primary tumor location in papillary thyroid carcinomas*

**DOI:** 10.3906/sag-1807-79

**Published:** 2019-02-11

**Authors:** Mehmet Eser SANCAKTAR, Güleser SAYLAM, Bülent ÖCAL, Ahmet ULUAT, Ömer BAYIR, Erman ÇAKAL, Mehmet Hakan KORKMAZ

**Affiliations:** 1 Department of Otolaryngology, Head, and Neck Surgery, Samsun Training and Research Hospital, Samsun Turkey; 2 Department of Otolaryngology, Head, and Neck Surgery, Dışkapı Yıldırım Beyazıt Training and Research Hospital, Ankara Turkey; 3 Department of Otolaryngology, Head, and Neck Surgery, Evliya Çelebi Training and Research Hospital, Kütahya Turkey; 4 Department of Endocrinology and Metabolism, Ministry of Health,Dışkapı Yıldırım Beyazıt Training and Research Hospital, Ankara Turkey; 5 Department of Otolaryngology, Head, and Neck Surgery, Yıldırım Beyazıt University, Faculty of Medicine, Ankara Turkey

## 1. Introduction

PTC constitutes approximately 80%–85% of all thyroid cancers with reported 10-year survival of >90% (1). The rate of metastases to the regional lymph nodes ranges from 50% to 80% in some series (2–4). PTCs generally metastasize to the CN more often than the LN (5)**.**

Lymph node metastasis may be a risk factor for recurrence and distant metastasis in PTC. CNM has poor prognostic value but increases locoregional recurrence (6). LNM also increases the risk of recurrence and distant metastasis (7). In addition to the effects of metastases on survival and recurrence, which are still open to discussion, the effects of many prognostic factors such as age, sex, primary tumor location, size of the tumor, extracapsular spread, and histopathology have become issues in current studies (1,6,7). In recent decades, clinical trials about the effects of primary tumor location on lymph node metastasis have provided conflicting results (1,6,7). PTC mostly presents a sequential lymph node metastasis pattern although a discontinuous lymph node metastasis pattern is also possible. Lee et al. (7) reported that primary tumors located in the upper pole of the thyroid gland are closely linked to skipped metastases in the lateral cervical neck.

The aim of this study was to investigate the predictive role of tumor location on central and lateral cervical lymph node metastasis pattern in PTCs.

## 2. Materials and methods

The study was conducted at Dışkapı Yıldırım Beyazıt Training and Research Hospital ENT Department after the approval of the Ethics Committe. A retrospective analysis was made of the medical records of 467 patients who underwent thyroid surgery between January 2008 and June 2012. The last preoperative USG reports, fine needle aspiration biopsy (FNAB) results, operation notes, and postoperative histopathological results were reviewed in detail. Patients were excluded if revision or complementary thyroid surgery was performed, if there were benign causes, or if other histological types of thyroid carcinoma were determined. Multifocal or multicentric tumors were also excluded. The patients in this study were only those who underwent total thyroidectomy (TT) plus either only central neck dissection (CND) or CND with lateral neck dissection (LND). CND was performed bilaterally to all patients whose FNAB results were malignant. LND was performed if lateral cervical lymph node metastasis was evident radiologically or confirmed by ultrasound-guided FNAB and routinely included neck levels 2, 3, and 4. Eventually, 110 PTC patients were included in the study.

### 2.1. Evaluation of localization 

The localization of the primary tumor was determined according to the last preoperative USG report which had been produced by the same team. It was checked from the reports if the nodules were described as in the lower pole, middle pole, upper pole, and isthmus. The descriptions in the horizontal plane such as back, front, lateral and medial settling patterns of the nodule were not taken into consideration. Tumors localized in the isthmus were evaluated as middle pole. The localization of the nodules on the USG reports was confirmed by the histopathology report.

### 2.2. Statistical analysis

Data were transferred from Excel files to IBM ISS 20 program and the analyses were completed. Before analysis, the conformity of continuous variables to normal distribution was assessed. If >50, the Kolmogorov–Smirnov test was applied and if <50, the Shapiro–Wilk test. As the age value conformed to the normality hypothesis in both groups, the independent sample t-test was applied in comparisons at 95% confidence interval. The Pearson Chi-square test was applied to categorical variables such as age, sex, tumor localization, side, and micropapillary and papillary presence, which were analyzed in respect of the metastasis effect to central and lateral compartments.

## 3. Results

The study included a total of 110 patients who underwent TT + CND and/or TT + CND + LND. According to the final pathology reports, PTC was determined in 65 patients (59%) and micropapillary carcinomas (mPTC) in 45 (41%). The patients comprised 96 (87.3%) females and 14 (12.7%) males with a mean age of 43.8 years (range, 19–85 years).

The effects of the variables of sex, tumor location, side, and metastasis to the CN and LN were investigated for both papillary and micropapillary carcinomas (Table 1). The surgery types were also determined. 

**Table 1:** Demographic data of patients according to the PTC and mPTC.

		PTC		mPTC	
		n	%	n	%
Sex	Female	55	84.6	41	91.1
	Male	10	15.4	4	8.9
Location	Lower pole	17	26.2	16	35.6
	Middle pole	33	50.7	27	60.0
	Upper pole	15	23.1	2	4.4
Side	Right lobe	31	47.7	21	46.7
	Left lobe	29	44.6	24	53.3
	Isthmus	5	7.7	0	0.0
Surgery	TT + CND	52	81.5	44	97.8
	TT + CND + LND	8	10.8	1	2.2
	TT+CND+BLND	5	7.7	0	0.0
Central metastasis	Absent	35	53.8	32	71.1
	Present	30	46.2	13	28.9
Lateral metastasis	Absent	52	80.0	44	97.8
	Present	13	20.0	1	2.2
Total		65	100	45	100

CND, central neck dissection; LND, lateral neck dissection; BLND, bilateral neck dissection; TT, total thyroidectomy.

Primary tumors located in the upper pole, metastasized to the central lymph nodes (level 6) at a lower rate (17.6%) than those located in the middle (40.0%) or lower (48.5%) poles (P = 0.104) (Table 2), and at a statistically significantly lower rate (13.3%) in the PTC group (P < 0.05) (Table 3).

**Table 2:** The relationship between location and metastasis for all patients.

Location		Lower (n=33)	Middle (n=60)	Upper (n=17)	Total (n=110)
Central metastasis (P = 0.104)	n	16	24	3	43
	%	48.5	40.0	17.6	39.1
Lateral metastasis(P = 0.326)	n	3	7	4	14
	%	9.1	11.7	23.5	12.7

**Table 3:** The relationship between location and metastasis in the PTC group.

Location		Lower (n=17)	Middle (n=33)	Upper (n=15)	Total
Central metastasis (P = 0.014)	n	10	18	2	30
	%	58.8	54.5	13.3	46.2
Lateral metastasis (P = 0.558)	n	2	7	4	13
	%	11.8	21.2	26.7	20.0

There was no significant relationship between the tumor side and central metastasis pattern in both PTC and mPTC patients.

### 3.1. Metastasis to lateral neck

Fourteen primary tumors metastasized to the LN (level 2-3-4). UPTs (n = 17) metastasized to the LN at almost twice the rate (23.5%) of middle (11.7%) or lower pole tumors (9.1%) (P = 0.326) (Table 2). Of 4 UPTs, 3 skipped to the LN without CNM (Table 4). In the mPTC patients, there was 1 LNM with CNM, located in the lower pole. Statistical analysis could not be performed due to the low numbers.

**Table 4:** The relationship between tumor location and lateral neck metastasis.

Localization	Central + Lateral	Lateral
Upper pole (n=17)	1	3
Middle pole (n=60)	5	2
Lower pole (n=33)	3	

## 4. Discussion

In PTCs, the first lymphatic station in the neck is the CN (level 6) and a high incidence of (up to 80%) occult metastases have been identified in the central compartment (2-4) . Metastasis to regional nodes is common in patients with PTC and has been reported to be associated with increased recurrence and compromised survival (8,9). 5-year survival rate is still reported with >99 % in PTCs excellently. Involvement of regional lymph nodes decrease this rate to 97 %, and in most cases is associated to higher regional recurrence rates (3,10).

Consensus has been reached on the decision for lymph node dissection if the central or lateral neck has evident lymph node metastasis (11). However, the discussions still continue for occult lymph node metastasis in clinical or radiological N0 necks. In many studies, it has been indicated that lymph node dissection has reduced recurrence and has been a positive effect on survival (12-14). 

Since the importance of lymph node metastases is now better understood, prognostic risk factors affecting the nodal metastasis pattern need to be taken into consideration in the decision for surgery. Metastases to the lateral neck have been found to have higher recurrence rates than metastases to the central compartment (15).

In a study by Wang et al. (1), it was concluded that tumors located in the middle and lower pole, tumors >0.5 cm in diameter, age <45 years and capsule invasion are high risks for metastases to the central region. 

Many authors researched the effect of intrathyroidal tumor location on the pattern of lymph node metastasis and concluded that tumors located in the upper pole tend to metastasize to the LN more frequently in PTC. It was emphasized that the LN must be examined carefully if the tumor is identified in the upper pole during the preoperative evaluation (6,16,17).

PTC mostly presents a sequential lymph node metastasis pattern, although a discontinuous lymph node metastasis pattern is also possible (6,7). Lee et al. (7) determined skip metastases to the LN in 9 patients where the primary tumor was located in the upper pole according to the ultrasonography findings. Ito et al. (18) determined that in PTC, tumor cells located in the upper pole tend to spread to the lateral lymph node throughout the superior thyroid artery. Zhang et al. (19) investigated the relationship between primary tumor location and nodal metastasis risk and concluded that upper pole location is a low risk for the central region but high risk for lateral region metastases. American Head and Neck Society Consensus mentioned PTC arising in the upper pole of the thyroid has a higher propensity to demonstrate skip metastases to levels III and II of the lateral compartment (20).

In the current series, CNM rates according to tumor location of upper, middle, and lower pole were 17.6%, 40.0%, and 48.5%, respectively, overall. Although not statistically significant, UPTs tended to CNM at a rate 2-fold lower and at a significantly lower rate (13.3%) in the PTC group than the mPTC group (P < 0.05). UPTs (n = 17) metastasized to the LN at a rate almost twice (23.5%) as that of middle (11.7%) or lower pole tumors (9.1%). It was also observed that 3 of 4 UPTs skipped to the LN without CNM.

Whether the primary tumor was in the right or left lobe in both the papillary and micropapillary groups had no effect on CNM. According to the current study data, because of the low number of metastases to the LN, the results were not as strong as in similar studies. However, these results support the findings of similar previous studies in the literature (1,6,7) since only 4 patients of 17 had upper pole-located primary tumor and 3 of those skipped metastasis to the LN. 

The major drawback that diminishes the reliability of these studies is that there is no standardization for the determination of primary tumor location, and that management of the neck differs locally in PTC (1,6,7). In the preoperative assessment, USG is accepted as the gold standard as it is inexpensive, noninvasive, and can be applied simultaneously with FNAB. However, it should be taken into consideration that results may change depending on the practitioner and specifying lymph nodes in the CN decreases the specificity and sensitivity (21). While the localization of solitary tumors is easier, determining the localization of multifocal or multicentric tumors presents a significant challenge. In this study, solitary PTCs according to pathology reports were included even if the patient was diagnosed as multinodular. Multifocal or multicentric tumors were excluded. Further clinical trials are required to investigate the predictors of the nodal metastasis pattern and these would be of benefit in planning surgery. If specific results can be obtained by subdividing the central region (paratracheal, pretracheal, prelaryngeal etc.), surgeries could be planned which would cause less morbidity.

In localization studies, including the current study, the relationship of primary tumor location and the papillary carcinoma histological subtypes has not been examined; thus, the effects of subtypes of PTC on the lymph nodes spread pattern can be considered to differ. In future studies evaluating the effect of location on the lymph node metastasis pattern, the effects of folliculary carcinoma, and medullary and anaplastic carcinoma should also be examined. 

In conclusion, we think that the lymph node metastasis pattern of primary tumors located in the upper pole of the thyroid gland differs from those with middle and lower pole localization. In our opinion, upper pole-located tumors have propensity to demonstrate metastasis to LN. We consider that UPTs should be evaluated more carefully preoperatively and routine CND should not be applied to these patients if there are no other prognostic risk factors. Predictive prognostic factors should be prospectively evaluated in wider patient series with more detailed identification of tumor localization, taking into account the histological subtypes with the specific and standardized methods.

## Acknowledgments

The authors would like to thank Associate Prof Cem Saka for his outstanding assistance and guidance in all working stages and thank all patients for access to their medical records. We also wish to thank to all members of Multidisciplinary Endocrine Tumor Board for their contribution in facilitating this and other endocrine surgical projects.
